# 5-Aminolevulinic Acid Improves Morphogenesis and Na^+^ Subcellular Distribution in the Apical Cells of *Cucumis sativus* L. Under Salinity Stress

**DOI:** 10.3389/fpls.2021.636121

**Published:** 2021-03-18

**Authors:** Yue Wu, Na Liu, Linli Hu, Weibiao Liao, Zhongqi Tang, Xuemei Xiao, Jian Lyu, Jianming Xie, Alejandro Calderón-Urrea, Jihua Yu

**Affiliations:** ^1^College of Horticulture, Gansu Agricultural University, Lanzhou, China; ^2^Department of Biology, College of Science and Mathematics, California State University, Fresno, Fresno, CA, United States; ^3^College of Plant Protection, Gansu Agricultural University, Lanzhou, China; ^4^Gansu Provincial Key Laboratory of Arid Land Crop Science, Gansu Agricultural University, Lanzhou, China

**Keywords:** salinity, 5-aminolevulinic acid, Na^+^ distribution, proton pump, Na^+^/H^+^ antiporter, root architecture

## Abstract

Soil salinity causes damage to plants and a reduction in output. A natural plant growth regulator, 5-aminolevulinic acid (ALA), has been shown to promote plant growth under abiotic stress conditions. In the present study, we assessed the effects of exogenously applied ALA (25 mg L^−1^) on the root architecture and Na^+^ distribution of cucumber (*Cucumis sativus* L.) seedlings under moderate NaCl stress (50 mmol L^−1^). The results showed that exogenous ALA improved root length, root volume, root surface area, and cell activity in the root tips, which were inhibited under salt stress. In addition, although salinity stress increased the subcellular Na^+^ contents, such as those of the cell wall, nucleus, plastid, and mitochondria, ALA treatment reduced these Na^+^ contents, except the soluble fraction. Molecular biological analysis revealed that ALA application upregulated both the SOS1 and HA3 transcriptional and translational levels, which suggested that the excretion of Na^+^ into the cytoplasm cloud was promoted by exogenous ALA. Meanwhile, exogenously applied ALA also upregulated the gene and protein expression of NHX1 and VHA-A under salinity stress, which suggested that the compartmentalization of Na^+^ to the vacuole was enhanced. Overall, exogenous ALA mitigated the damage caused by NaCl in cucumber by enhancing Na^+^ redistribution and increasing the cytoactivity of root cells.

## Introduction

High salinity levels in cultivated fields are caused by a combination of factors, such as excessive chemical fertilizer application, prolonged cultivation, or the effects of global climate change. Globally, almost 30 plant species provide 90% of the food consumed by the human population (Zörb et al., 2019). However, most of these species are salt-sensitive, and more than 20% of crops are affected by soil salinity (Negrão et al., 2016). Therefore, ways to improve the salt tolerance of crops has received a recent increase in research attention among agricultural scientists.

The imbalance of the K^+^/Na^+^ ratio under salt stress conditions adversely affects plant growth (Shah et al., 2021). Specifically, the harmful effects of high Na^+^ concentration in the cytoplasm leads to ion toxicity, which can be observed at the whole-plant level, as lower growth rates and leaf and root damage. Under saline conditions, extracellular Na^+^ competes with the K^+^ transport in cells because Na^+^ and K^+^ have a similar hydrated radius (Gong et al., 2010). The flow of Na^+^ into cells is a passive process, which requires a negative electric potential difference across the plasma membrane. However, the displacement of Na^+^ is an active process that involves Na^+^/H^+^ antiporters located on the plasma membrane and tonoplast, which regulate the compartmentation and efflux of Na^+^ (Yamaguchi et al., 2013). The Na^+^/H^+^ antiporters on the plasma membrane and tonoplast are encoded by *salt overly sensitive 1* (*SOS1*) and Na^+^/H^+^ exchanger (*NHX1*), respectively (Zhang et al., 2017; Tanveer, 2020). The cytoplasmic enzymes of halophytes are as sensitive to salinity levels as the enzymes of glycophytes (Wakeel et al., 2011). Therefore, to alleviate the ion toxicity, Na^+^ can be compartmentalized into vacuole or excreted out of protoplast. The adaption of halophilous plants in saline conditions is due to their active and efficient reduction of cytoplasmic Na^+^ levels, and their ability to maintain relatively lower Na^+^ concentrations and higher K^+^/Na^+^ ratios in the cytoplasm (Cuin et al., 2011).

Ion imbalance induced by salinity directly affects plant growth in the rhizosphere environment. Under saline conditions, the Na^+^ and K^+^ contents have been shown to sharply increase and decrease in root cells, respectively (Zhang et al., 2018; Huo et al., 2020). Additionally, salt stress negatively affects the lateral root number and total root length, which leads to an incomplete root system architecture (Julkowska et al., 2017). Meanwhile, H^+^-pumps and Na^+^/K^+^ transporters, which contribute to cell ion exchange and homeostasis, respond (i.e., are up- or downregulated) to high Na^+^ conditions as physiological adaptations (Zhao et al., 2016; Yan et al., 2020). In addition, lipid peroxidation occurs in root cells; thus causing increases in reactive oxygen species (Nath et al., 2019) and a reduction in root cell activity (Wang et al., 2020).

5-Aminolevulinic acid (ALA) is a metabolic intermediate in plants, animals, and bacteria. The downstream metabolic pathway of ALA provides chlorophyll, heme, siroheme, vitamin B_12_, and phytochromobilin in higher plants (Wu et al., 2019b). ALA has been considered to play a promotive role in plants under abiotic stress and normal conditions (Ali et al., 2013a,b). Under saline conditions, the gene expression levels of proline synthesis can be upregulated by ALA in *Brassica napus* L. seedlings (Xiong et al., 2018). Moreover, exogenous ALA was shown to regulate the accumulation of H_2_O_2_ in roots to activate the relative gene expression of Na^+^ transporters, which mitigated the harmful effect of salt on the shoots (Wu et al., 2019a). Our previous study showed that exogenous ALA enhanced the photosynthesis of cucumber leaves under NaCl stress by upregulating chlorophyll biosynthesis (Wu et al., 2018). In this study, we conducted physiological and molecular level investigations to explore the promotive role of ALA on cucumber roots under saline (NaCl) rhizospheric conditions. Further, we also investigated the root architecture, ultrastructure of apical cells, and Na^+^ distribution of cucumber root cells, and the mechanism by which ALA application impacted these factors.

## Materials and Methods

### Plant Material and Growth Conditions

Cucumber (*Cucumis sativus* L. ‘Xinchun No. 4’) seedlings were used as the plant material. Healthy plump seeds were selected and sterilized with 0.03% potassium permanganate solution for 10 min. After soaking for 6 h in distilled water, seeds were placed on wet filter paper under dark conditions (28 ± 1°C). After germination, seedlings were grown in a climatic cabinet for 5 days, with a light intensity of 350–450 μmol m^−2^ s^−1^, photoperiod 12/12 h, temperature of 28/18°C (day/night), and relative humidity of 50–60%. Seedlings with fully spread cotyledons, of uniform size, and with healthy roots were selected and transferred to opaque plastic containers for hydroponic culturing. Each container contained four cucumber seedlings. During plant cultivation, the half-strength Yamasaki cucumber nutrient solution was used [Ca(NO_3_)_2_ 1.75 mmol L^−1^, KNO_3_ 3 mmol L^−1^, NH_4_H_2_PO_4_ 0.5 mmol L^−1^, and MgSO_4_·7H_2_O 1 mmol L^−1^]. The nutrient solution was changed at 2-day intervals. The experimental treatments were executed using seedlings that had been grown for 30 days.

### Experimental Design

Cucumber seedlings with three fully expanded true leaves, uniform size, and healthy roots were selected for the treatments. Four treatments were used in this study: (1) Control: normal nutrient solution; (2) NaCl: 50 mmol L^−1^ NaCl in nutrient solution; (3) NaCl + ALA: 50 mmol L^−1^ NaCl in nutrient solution + 25 mg L^−1^ foliar sprayed ALA, and (4) ALA: normal nutrient solution + 25 mg L^−1^ foliar sprayed ALA. The concentrations of moderate NaCl stress and optimal ALA application were selected based on the findings of our previous study (Wu et al., 2018). ALA (Sigma Aldrich, United States) applications were applied by thoroughly spraying the prepared solution on both the upper and lower surfaces of leaves with a hand sprayer (200 ml per treatment). Moreover, ALA applications involved two treatments, i.e., at 0 and 24 h. In the treatments that did not receive ALA applications, distilled water was sprayed in the same manner. Each replication comprised 10 containers, and each treatment was replicated three times. Experimental treatments were arranged in a completely randomized design. The nutrient solution was changed at 2-day intervals and aerated continuously with an air pump. All indexes were measured at 10 days after treatment application.

### Root Morphology Indexes

The roots were removed from the seedlings and rinsed with distilled water. The root architecture was photographed using a root scanner (STD 4800, Canada), and the root morphology indexes (including total root length, root volume, root surface area, and number of root tips) were analyzed using Win RHIZO 5.0 (Regent Instruments, Inc., Canada).

### Root Activity and Cell Viability Staining

For fluorescence microscopy, fluorescein diacetate (FDA; 5 mg; Sigma Aldrich, United States) was dissolved in 1 ml acetone. The FDA solution (0.4 ml) was then made up to volume (5 ml) with 0.65 mmol L^−1^ mannitol. Propidium iodide (PI; 2 mg; Sigma Aldrich, United States) was dissolved in 0.65 mmol L^−1^ mannitol, and made up to volume of 5 ml. The FDA and PI solutions were mixed in equal volumes, and the root tips were soaked in the mixed solution for 40 min in the dark, and then washed three times with deionized water for 5 min each. Red and green fluorescence and concurrent images were obtained with a fluorescent microscope (Leica DM600 400×, Germany) at 485 and 530 nm, excitation and emission, respectively. The fluorescence densities of the FDA and PI solutions were analyzed using ImageJ software.

Root activity was determined according to the method of Zhang et al. (2013). Root tip samples (0.5 g) were soaked in a solution containing 5 ml 0.4% (w/v) triphenyl tetrazolium chloride (TTC) and 5 ml PBS (pH 7.0) under dark conditions for 1 h at 37°C. Sulfuric acid (2 ml, 1 mol L^−1^) was then added to end the reduction of TTC. Root samples were ground with 5 ml of acetic ether and quartz sand. The extracted solution from the root was fixed to 10 ml with acetic ether. Absorbance was measured at 485 nm, and the root activity was calculated according to the standard curve.

### Contents of Na^+^, K^+^ in Root Subcellular Fraction

The subcellular fractions of cucumber roots were separated according to the method described by Jabeen et al. (2014). Root samples were fully homogenized with 5 ml extracting solution (0.25 mol L^−1^ sucrose, 50 mmol L^−1^, pH 7.5 Tris-HCl, and 1 mmol L^−1^ dithioerythritol), and then filtered through a filter cloth (80 μm). The filter residue was the cell wall fraction. The filtrate was made up to volume (40 ml) with the extraction solution mentioned previously. The solution was centrifuged at 1,500 × *g* for 10 min to obtain the sediment (the plastid fraction). The supernatant was then centrifuged at 5,000 × *g* for 20 min to obtain the nucleus fraction, and again at 15,000 × *g* for 30 min to obtain the mitochondrial fraction. The final supernatant represented the soluble fraction of the root cell. Every subcellular fraction was dried in an oven, and the Na^+^ and K^+^ contents were determined using an atomic absorption spectrometer (ZEEnit 700P, Analytik Jena, Germany).

### Fluorescence Microscopy of Na^+^ on Root Tip and Hypocotyl

The fluorescence of Na^+^ was observed according to the method of Bonales-Alatorre et al. (2013). Coro-Na™-Green AM (Invitrogen, Thermo Fisher Scientific, United States) was assisting dissolved by 0.2 ml dimethyl sulfoxide. Samples with four repetitions in each treatment were stained with 15 μmol L^−1^ CoroNa™-Green AM, which was dissolved by Ca^2+^-MES (pH 6.1). Root tip and hypocotyl of seedlings were length cut and stained in dark condition for 1 h and then washed three times with deionized water for 5 min each. The green fluorescence of Na^+^ and images were obtained with a fluorescent microscope (Leica DM600 400×, Germany) at 488 and 525 nm, excitation and emission, respectively. The relative green fluorescence intensity of Na^+^ revealed in root or hypocotyl was analyzed using ImageJ software, which indicated the light intensity of in unit area of each sample.

### Western Blot Analysis

The protein expression levels of Na^+^/H^+^ antiporter in plasma membrane (SOS1), Na^+^/H^+^ antiporter in tonoplast (NHX1), H^+^-ATPase in plasma membrane (HA3), and H^+^-ATPase in tonoplast were analyzed by Western blotting. The membrane protein of cucumber plants was extracted using the TCA/acetone method. The concentration of protein samples was measured using the bicinchoninic acid (BCA) method and a BCA Protein Assay Kit (Beyotime Biotechnology, P.R. China). Membrane protein samples were separated by SDS-PAGE. The separated proteins were transferred onto a polyvinylidene difluoride (PVDF) membrane, and the nonspecific binding of antibodies was blocked with 5% non-fat dried milk in PBS for 1 h at 25 ± 1°C. Membranes were then incubated overnight at 4°C with polyclonal antibodies at the appropriate dilution against SOS1 (1:2500; Agrisera, Vännäs, Sweden), NHX1 (1:2500; Agrisera, Vännäs, Sweden), HA3 (1:2500; Agrisera, Vännäs, Sweden), and VHA-A (1:5000; Abcam, Shanghai, China). The PVDF membrane was then incubated with goat anti-rabbit IgG (H&L) and HRP-conjugated secondary antibody (diluted 1:3000) for 1 h at room temperature. The color was developed using an electrochemiluminescence (ECL) substrate (BioRad, United States). Finally, the developed films were scanned and precisely quantified using Amersham Imager 600 (General Electric, United States).

### Real-Time qPCR Analysis

The gene transcriptional levels of *SOS1*, *NHX1*, *HA3*, and *VHA-A* were determined by q-PCR. The cucumber *U6* gene was used as an internal control. The gene bank accession numbers of the sequences used to design the primers are shown in Table 1. Treatment samples were randomly obtained from three roots, and each treatment was replicated three times. The TaKaRa MiniBEST Plant RNA Extraction Kit (TaKaRa Biomedicals, Japan) was used to extract the total RNA from cucumber roots. The cDNA was synthesized using the Revert Aid First Strand cDNA Synthesis Kit (Thermo Scientific, United States). The reaction system of the PCR test consisted of 2 μl cDNA, 0.8 μl forward primer, 0.8 μl reverse primer, 10 μl 2 × Tli RNaseH Plus, and 6.4 μl RNase Free dH_2_O. The PCR procedure was executed for three technical replications per biological sample. The PCR reaction conditions were as follows: first, 95°C for 30 s for the initial denaturation, and then 95°C for 5 s, 60°C for 30 s for the cycle steps (40 cycles), 95°C for 5 s, 60°C for 60 s for the melting curve, and, finally, 50°C for 30 s for the cooling step. Quantification analyses were conducted using the comparative CT value method, following Livak and Schmittgen (2001).

**Table 1:** Primer sequences and Genbank accession numbers of *SOS1*, *NHX1*, *HA3*, *VHA-A*, and *U6* gene.

Gene symbol	Accession number	Forward primer	Reverse primer
SOS1	JQ655747.1	5'-AGGAAGGTTCAAAGCCTAGTG-3'	5'-CATGAGTAAATGTGGGGTGCA-3'
NHX1	FJ843078.1	5'-TGCTTTTGCCACCCTTTCA-3'	5'-TTCCAACCAGAACCAATCCC-3'
HA3	EF375892.2	5'-TGGAAAACAAGACCGCCTTT-3'	5'-GGTTGGAGGCCATGTAAGGTT-3'
VHA-A	AY580162.1	5'-CATTCCTGGAGCGTTTGGTT-3'	5'-CATTTCATTTCCTCTCTCTCCACAA-3'
U6	JW929310.1	5'-ACAGAGAAGATTAGCATGGCC-3'	5'-GACCAATTCTCGATTTGTGCG-3'

### Ultrastructure Observation

Fresh root tip samples (0.5 cm) were fixed in 3% glutaraldehyde in 0.1 mol L^−1^ phosphate buffer (pH 7.4) for 48 h at 4°C, and then fixed in 1% H_2_OsO_4_ solution for 5 h. Samples were then dehydrated using a graded ethanol series (70, 80, 90, and 100%) and then embedded in Epon812 epoxy resin. Ultrathin slices were cut using a microtome (Leica EM UC6 ultra-microtome, Japan), and the tissue slices were stained with uranyl acetate and lead citrate for 15 min. Ultrathin tissue slices of cucumber roots were observed and photographed using a transmission electron microscope (TEM, Joel JEM-1230, Japan).

### Statistical Analysis

Analyses of variance were performed using SPSS 22.0 (SPSS Institute Inc., United States) software, and the treatment means were compared using Tukey’s test, at a 0.05 level of probability. The results are expressed as means ± SEs. All figures were prepared using OriginPro2017 (OriginLab Institute Inc., United States) software.

## Results

### ALA Treatment Restored Root Morphology Changes Associated With Salt Stress

**Figure 1:**
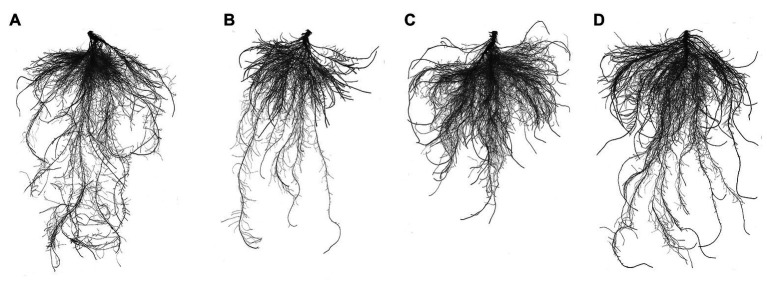
Root architecture characteristics of cucumber seedlings. **(A)** Normal growth condition. **(B)** Treatment with 50 mmol L^−1^ NaCl. **(C)** Treatment with 50 mmol L^−1^ NaCl + 25 mg L^−1^ ALA. **(D)** Treatment with 25 mg L^−1^ ALA under normal growth condition.

Under salt stress, the total root length, number of root tips, root volume, and root surface area decreased by 33.96, 46.13, 58.17, and 47.46%, respectively (Table 2 and Figure 1). However, exogenous application of ALA restored root growth to the level observed in the control group. Furthermore, the total root length was significantly improved by ALA under conditions without salt stress.

**Table 2:** Effects of exogenous 5-aminolevulinic acid (ALA) on root morphology parameters of cucumber seedlings under salt stress.

Treatment	Total root length (cm plant^−1^)	Root tips number (# plant^−1^)	Root volume (cm^3^ plant^−1^)	Root surface area (cm^2^ plant^−1^)
Control	2067.72 ± 130 b	1,589 ± 117 a	4.59 ± 0.43 a	345.38 ± 27 a
NaCl	1365.53 ± 3 c	856 ± 62 b	1.92 ± 0.09 b	181.45 ± 4 b
NaCl+ALA	1989.07 ± 98 b	1,616 ± 239 a	4.64 ± 0.96 a	337.54 ± 42 a
ALA	2426.27 ± 64 a	1,116 ± 106 ab	6.09 ± 0.33 a	430.46 ± 10 a

Value (mean ± SE) was the mean of three independent experiments, and significant differences (*p* < 0.05) among different treatments were indicated by different letters.

### ALA Treatment Abrogated Root Cell Activity Changes Associated With Salt Stress

**Figure 2:**
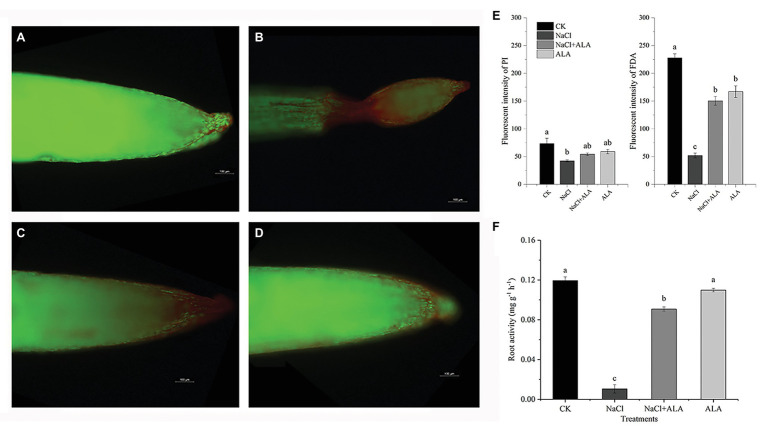
The living-death cell fluorescence and activity of apical cells of cucumber seedlings. **(A–D)** Viable cells show green fouorescence while non-viable cells show red. **(E)** Fluorescence intensities of green and red fouorescence. **(F)** Root tip activities of cucumber seedlings. Data represent means of three replicates. Bars indicate the SE. Significant differences (*p* < 0.05) between treatments are indicated by different letters.

The results of the living-dead fluorescence analysis of root cells are shown in Figures 2A–D. The control group root cells showed relatively higher green fluorescence. Red fluorescence was enhanced on the root tip under NaCl stress, especially in the apical elongation zone, which was wilted. The root tips of seedlings treated with ALA under salt stress showed normal apical morphology; however, the green fluorescence was enhanced. NaCl stress significantly decreased the intensities of FDA and PI. However, application of ALA markedly increased the fluorescence of living cells (Figure 2E). The root cell activity was suppressed under saline conditions; however, the application of ALA reversed the inhibitory effect of NaCl and significantly enhanced the root tip activity (Figure 2F).

### ALA Treatment Ameliorated Root Subcellular Na^+^, K^+^ Changes Associated With Salt Stress

**Figure 3:**
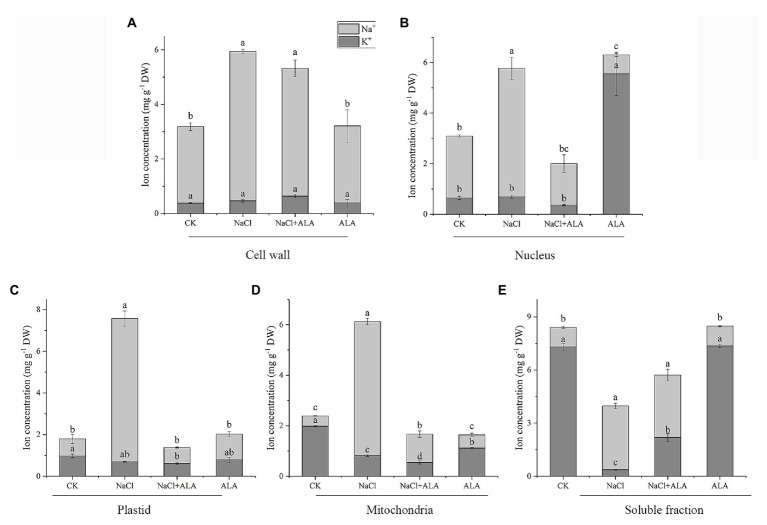
Subcellular Na^+^, K^+^ concentration in apical cells of cucumber seedlings. **(A)** Na^+^, K^+^ concentration in cell wall. **(B)** Na^+^, K^+^ concentration in nucleus. **(C)** Na^+^, K^+^ concentration in plastid. **(D)** Na^+^, K^+^ concentration in mitochondria. **(E)** Na^+^, K^+^ concentration in soluble fraction of cucumber root cells. Data represent means of three replicates. Bars indicate the SE. Significant differences (*p* < 0.05) between treatments are indicated by different letters.

After treatment with NaCl, the Na^+^ contents in the root cells increased significantly (Figure 3). The Na^+^ concentrations in the cell wall, nucleus, plastid, mitochondria, and soluble fractions of roots were 1.6-, 2.1-, 8.4-, 13.0-, and 3.3-fold higher than those of control group. Moreover, the K^+^ concentration was significantly decreased in the mitochondrial and soluble fractions. After the foliar spray of ALA, the Na^+^ contents in each of the root cell fractions were reduced significantly, except for the cell wall. Meanwhile, the K^+^ contents of the soluble fractions were markedly increased by exogenous ALA application under salt stress. Under normal growth conditions, the application of ALA increased the K^+^ content in the nucleus, but decreased its content in mitochondria, compared with the control.

### ALA Treatment Suppressed Na^+^ Fluorescence Changes Associated With Salt Stress

**Figure 4:**
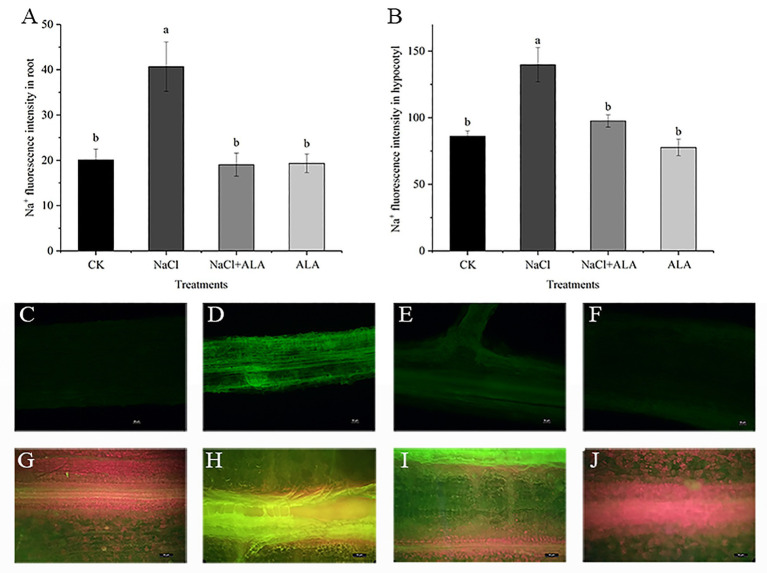
The fluorescence images of Na^+^ in root and hypocotyl of cucumber seedlings. **(A)** Fluorescence intensities of Na^+^ in root. **(B)** Fluorescence intensities of Na^+^ in hypocotyl. Data represent means of four replicates. Bars indicate the SE. Significant differences (*p* < 0.05) between treatments are indicated by different letters. **(C–F)** The Na^+^ fluorescence images in root of seedlings. Scale bar = 50 μm. **(G–J)** The Na^+^ fluorescence images in hypocotyl of seedlings. Scale bar = 50 μm.

The Na^+^ absorption of cucumber root tips and hypocotyls were determined by staining with CoroNa™-Green AM (Figure 4). Under NaCl stress, the Na^+^ intensity in the root tips and hypocotyls increased by 102.39 and 62.41%, respectively, compared with the control (Figures 4A,B). The Na^+^ fluorescence in the root tissue was obviously enhanced, especially in the epidermis and stele. However, the fluorescence intensity in both the roots and hypocotyls decreased to the level of the control after exogenous application of ALA on the leaves. At the same time, the green fluorescence in the stele of the roots and hypocotyls was weakened (Figures 4C–J).

### ALA Treatment Upregulated Ion Transporters Levels Associated With Salt Stress

**Figure 5:**
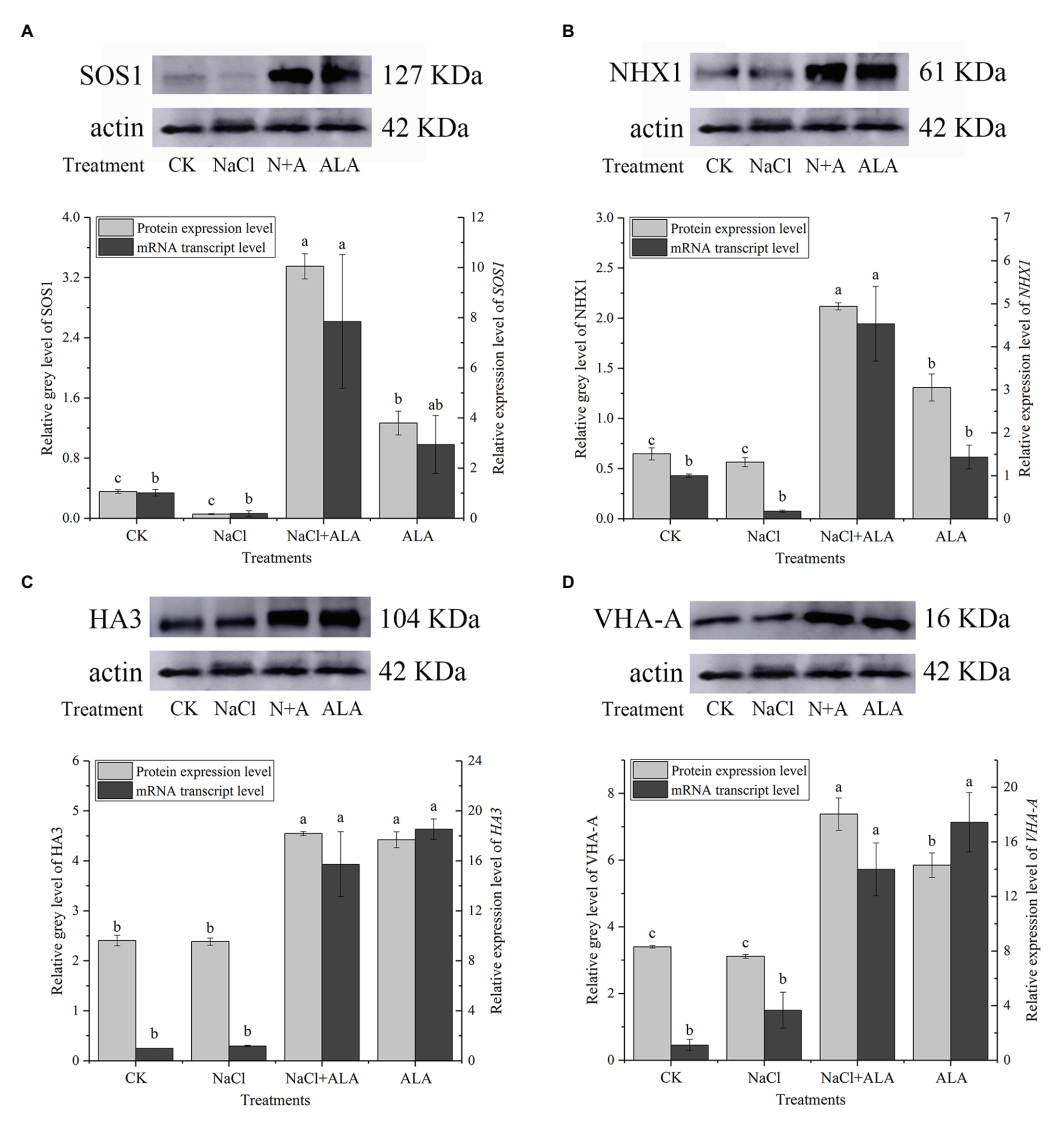
The protein and gene expression levels of ion transporters in cucumber root. **(A)** Protein/gene expression of Na^+^/H^+^ antiporter in plasma membrane (*SOS1*). **(B)** Protein/gene expression of Na^+^/H^+^ antiporter in tonoplast (*NHX1*). **(C)** Protein/gene expression of H^+^-ATPase in plasma membrane (*HA3*). **(D)** Protein/gene expression of H^+^-ATPase in tonoplast (*VHA-A*). The bands are the protein expression bands of each transporter protein, and the histograms showing the ratio of gray level to actin gray level of ion transport protein expression. Data represent means of three replicates. Bars indicate the SE. Significant differences (*p* < 0.05) between treatments are indicated by different letters.

The expression of SOS1 protein was downregulated under saline conditions but was significantly enhanced by exogenous ALA (Figure 5A). Meanwhile, the protein expression of SOS1 was also upregulated by ALA alone. Under salt stress, the relative expression level of *SOS1* was downregulated, but not significantly, compared to the control. The application of ALA significantly upregulated the transcriptional level of *SOS1* under salt stress conditions, i.e., to 7.8 times that of the control. The protein expression level of the tonoplast Na^+^/H^+^ transporter, NHX1, remained stable under NaCl conditions; however, exogenous ALA stimulated its expression to increase by 3-fold, compared to the control (Figure 5B). Moreover, the expression of *NHX1* was upregulated by ALA under normal conditions and under NaCl stress conditions (i.e., by 4.5-fold compared to the control). However, the results of the other treatments did not differ significantly from those of the control. The investigation of the proton pump in cucumber root cells revealed that the protein expression levels of HA3 and VHA-A remained stable under salt stress. However, exogenous ALA significantly upregulated their expression under stressful and non-stressful conditions (Figures 5C,D). Similar to *SOS1*, exogenous ALA enhanced the *HA3* gene expression level to 15 times under NaCl conditions when compared with the control (Figure 5C). However, the relative expression of the *VHA-A* gene was significantly upregulated by exogenous ALA under both salt stress and non-stress conditions; i.e., were 13 and 17 times that of the control, respectively (Figure 5D).

### ALA Treatment Improved Apical Cells Ultrastructure Changes Associated With Salt Stress

**Figure 6:**
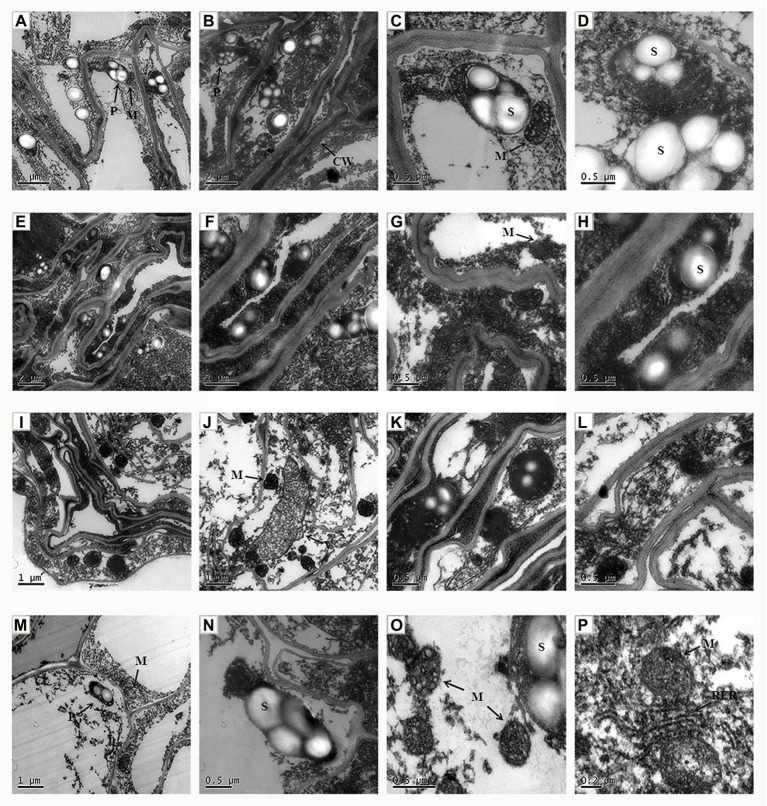
The ultrastructural observation of root tip cell of cucumber seedlings. **(A–D)** Seedlings grown in control condition. **(E–H)** Fifty millimole per liter NaCl treated seedlings. **(I–L)** Seedlings treated with 50 mmol L^−1^ NaCl and 25 mg L^−1^ ALA simultaneously. **(M–P)** Seedlings sprayed 25 mg L^−1^ ALA only. CW, cell wall; P, plastid; S, starch; M, mitochondria; V, vacuole; RER, rough surfaced endoplasmic reticulum.

The ultrastructure of the apical cell was observed by transmission electron microscopy. In the control group, the apical cells showed a regular morphology, with a clear central vacuole, a normal number of plastids, and sufficient starch granules. The mitochondrial morphology was regular, with a clear intimal structure and they gathered near the plastids (Figures 6A–D). However, under NaCl stress the cell morphology of root cells was obviously altered (Figures 6E–H). The cell wall became thicker and had a wave shape, and the number of plastids and starch decreased; however, the number of mitochondria increased. In addition, dark ion deposition increased in the cytoplasm. Exogenous application of ALA under salt stress reduced the ion deposition in the cytoplasm of apical cells, and the central vacuole was clearly visible (Figures 6I–L). However, the cell morphology was not obviously improved, the intercellular space was enlarged, the plastids and starch granules were rare, and the number of mitochondria increased. Moreover, when ALA was applied under control conditions, the cell morphology was regular; the mitochondria gathered near the plastids, and clear rough endoplasmic reticuli were visible (Figures 6M–P).

## Discussion

Physiological damage occurs when plants suffer under saline conditions in the rhizosphere environment. Salt stress inhibits many physiological and biochemical responses, including the anatomical morphology of leaves, physiological function of cells, photosynthesis reactions, water transport, protein synthesis, and energy production (Khan et al., 2020). In recent years, a natural plant growth regulator, ALA, has frequently been reported to improve plant resistance to environmental stress (see Akram and Ashraf, 2013 for a review). In the present study, NaCl stress inhibited the growth of cucumber roots. Moreover, all root morphological parameters showed significant decreases, the lateral roots were sparse, and the length of the roots decreased significantly. Moreover, NaCl caused apoptosis and wilting and a significant reduction in the living cells of roots, especially in the apical elongation area of seedlings under salt stress. These findings are consistent with those of a study on *Arabidopsis thaliana* L., in which the activity of *Arabidopsis* apical cells decreased gradually after exposure to low pH conditions, and the area of dead cells expanded gradually. The apical elongation region of *A. thaliana* was completely apoptotic after 2 h of low pH stress (Koyama et al., 2001). However, exogenous ALA has been shown to positively affect the root inhibition caused by various abiotic stresses. For example, medium Cd stress was previously shown to inhibit the root growth of *B. napus*, but 25 mg L^−1^ exogenous ALA application significantly improved the root average diameter, total volume, and total superficial area (Ali et al., 2013b). Additionally, in the present study, ALA application significantly increased root cell activity and the number of living cells in the apical elongation zone. Similarly, in alfalfa (*Medicago sativa* L.) plants, hydrogen-rich water (10%, v/v) was shown to relieve the harmful effects of Cd stress and decrease the number of dead root cells (Cui et al., 2013).

A relatively higher rhizospheric salt condition can cause osmotic stress in plants. In the present study, Na^+^ was mainly enriched in the cell wall under normal growth conditions, while high K^+^ and low Na^+^ environments were found in plastids, mitochondria, and the soluble fractions of root cells. In contrast, a reduction in K^+^ has been observed in the plastids, mitochondria, and the soluble fractions of root cells under saline conditions. Moreover, in the present study, Na^+^ increased significantly in each fraction of the root cells under salt stress. Similarly, the Na^+^ concentration reportedly increased and the K^+^ concentration decreased in barley (*Hordeum vulgare* L.) under 300 mmol L^−1^ NaCl (Jabeen et al., 2014). Rhizospheric salt stress is considered to affect the ion balance in root cells and increase the Na^+^/K^+^ ratio. This imbalance occurs because Na^+^ and K^+^ have similar ionic radius, and excessive Na^+^ in the apoplast competes with the potassium channel. Furthermore, Na^+^ in trophoplasts disturbs the function of K^+^ and destroys the stability of the cell wall and cell membrane structure (Yamaguchi et al., 2013). In addition, excessive Na^+^ can cause damage to cell microtubules and ribosomes, and accelerate cell senescence (Munns, 1993). Therefore, maintaining the intracellular K^+^ content above a certain threshold and maintaining a high ratio of cell fluid K^+^/Na^+^ (i.e., retaining a higher K^+^ content, or preventing the accumulation of Na^+^ in leaves) is the key to normal growth and salt tolerance in plants under salt stress. Moreover, in this study, exogenous ALA reduced the accumulation of Na^+^ in cucumber root cells under salt stress and significantly lowered the Na^+^ level in organelles.

5-Aminolevulinic acid application was also shown to upregulate the transcriptional level under salt stress and the protein expression level of the Na^+^/H^+^ antiporter (SOS1) as well as the proton pump (HA3) on the plasma membrane. On the plasma membrane, the proton-motive force is mainly formed by a proton pump, and the proton-motive force is the major driving force for the transmembrane active transport of mineral elements in plant cells. The plasma membrane Na^+^/H^+^ antiporter is an electrically neutral Na^+^/H^+^ (1:1) transmembrane transporter that is dependent on the transmembrane H^+^ concentration gradient. Generally, the potential difference in the plasma membrane could be enhanced by increasing the salt concentration in the extracellular environment. At this time, the H^+^-ATPase on the plasma membrane could utilize the energy generated by the hydrolyzed ATP to pump H^+^ out of the cells to produce an electrochemical potential gradient. This force has the ability to activate the Na^+^/H^+^ antiporter, which can transport Na^+^ out of the cell against the electrochemical potential gradient (Allakhverdiev et al., 1999). The gene encoding the Na^+^/H^+^ antiporter was first investigated and cloned in *A. thaliana* and has been shown to be associated with the salt tolerance of plants (Shi et al., 2000). Compared with wild type plants, *Arabidopsis* plants with an overexpression of *SOS1* showed significantly decreased Na^+^ contents in the transpirational flow of xylem under saline conditions. Moreover, the calluses regenerated from transgenic plants also showed a strong tolerance to salt, and the concentration of Na^+^ in the cells was lower than that in wild type plants (Shi et al., 2002). In addition, transgenic *Arabidopsis* plants with the soybean *GmsSOS1* gene showed improved salt tolerance and reduced reactive oxygen species contents under salt stress, and the yeast cells expressing *GmsSOS1* showed a decrease in Na^+^ accumulation under NaCl conditions (Zhao et al., 2016). These research results suggest that the enhancement of the plasma membrane Na^+^/H^+^ antiporter and H^+^-ATPase to exude Na^+^ out of the cell are prerequisites for improving plant salt tolerance. Moreover, the Na^+^/H^+^ antiporter and H^+^-ATPase were enhanced by exogenous ALA in cucumber root cells under NaCl stress. In fact, the SOS1 protein functions through the SOS signal pathway, which is involved with two other proteins: SOS2 and SOS3, i.e., a protein kinase and a Ca^2+^-binding protein, respectively. Reportedly, Ca^2+^ can rapidly accumulate in the cytoplasm under high Na^+^ conditions, and a higher concentration of Ca^2+^ will activate SOS3. The activated SOS3 then binds SOS2 to form a SOS2-SOS3 calcium-dependent protein kinase complex, which can deactivate the self-inhibitory sites at the C terminal of the SOS1 protein by phosphorylation, and finally increase the activity of SOS1 (Liu and Zhu, 1998; Quintero et al., 2011).

Under saline conditions, Na^+^ is usually excreted out of the cells of glycophytes, but accumulates in the vacuoles of halophytes (Blumwald, 2000). Mineral ions in the cytoplasm, such as Na^+^, can be compartmentalized into vacuoles by ion transporters located on the tonoplast. There are two kinds of tonoplast proton pumps in plants: V-H^+^-ATPase and V-H^+^-PPase. These pumps hydrolyze ATP and PPi to pump H^+^ into the vacuole and develop proton-motive force across the vacuolar membrane, which stimulates the Na^+^/H^+^ antiporter on the tonoplast. For example, salt stress was shown to upregulate the proton pump and Na^+^/H^+^ antiporter on the tonoplast of *Suaeda salsa* L. leaves at the transcriptional level and transferred more Na^+^ into vacuoles (Qiu et al., 2007). Previously, transgenic *Arabidopsis* plants with the gene encoding the subunit B of the tonoplast H^+^-ATPase (*HcVHA-B*) of *Halostachys caspica* had significantly higher Na^+^ contents but an improved morphology and salt tolerance compared to the wild type plants, because of a substantial Na^+^ enrichment of the vacuoles (Hu et al., 2012). In the present study, the transcriptional and translational levels of the subunit A of V-H^+^-ATPase under NaCl stress did not show any significant difference compared to the control; however, exogenous ALA upregulated the gene and protein expression levels of VHA-A. This indicated that ALA application can improve the compartmentation of Na^+^ into vacuoles and enhance the salt resistance of cucumber seedlings. In fact, the tonoplast H^+^-ATPase is a multisubunit complex. Similarly, it was found that the subunit E of V-H^+^-ATPase, in *Broussonetia papyrifera* L., responded to relatively high salt conditions, and its mRNA and protein levels were upregulated under 150 mmol L^−1^ NaCl stress (Zhang et al., 2012). Overexpression of the subunits of V-H^+^-ATPase of salt-resistant wheat in *Arabidopsis* plants, including a, A, c, C, d, D, F, I, G, and H, was found to enhance the salt tolerance of transgenic plants. Among them, the most salt-tolerant was the transgenic *A. thaliana* with the c subunit (He et al., 2014).

The tonoplast Na^+^/H^+^ antiporter is a structural or constitutive protein whose activity is regulated by the Na^+^ content in halophytes (Apse and Blumwald, 2007). The activity of the tonoplast Na^+^/H^+^ antiporter can be affected rapidly by NaCl stress. In the presence of an NHX protein synthesis inhibitor, its activity can still be rapidly stimulated by NaCl, which might be due to the presence of NHX proteins rather than resynthesized proteins (Blumwald, 2000). Moreover, when salt-treated plants were transferred to Na-deficient conditions, the Na^+^/H^+^ antiporter was inactivated (Garbarino and DuPont, 1989). Interestingly, in *Plantago* species, the activity of the tonoplast Na^+^/H^+^ antiporter was only found in salt-tolerant *Plantago maritima* L., but not in salt-sensitive *Plantago media* L. (Staal et al., 1991). In this study, the seedlings treated with exogenous ALA showed higher mRNA and protein levels of NHX1, indicating that the capacity of Na^+^ compartments in cells can be improved with ALA application. The Na^+^, compared to the tonoplast Na^+^/H^+^ antiporter from the cytoplasm to the vacuole, can be used as an osmotic agent to maintain the cell osmotic potential and contribute to water absorption. In addition, in transgenic tomato plants expressing *SbNHX* of *Sorghum bicolor*, the tonoplast Na^+^/H^+^ antiporter was found to have an interactive relationship with the gene family of the cation/proton antiporter (*CPA*), such as *SOS*. This suggests that NHX proteins might assist the cation antiporters on the plasma membrane to excrete Na^+^ from cells and, thus, enhance the salt resistance of plants; however, the mechanism of their interaction remains unclear (Palavalasa et al., 2017). Besides, decrease of Na^+^ in cytoplasm could lead to cut down of ROS and alleviate of stress damages. For example, overexpressing transcriptional activator *NAC1* could significantly reduce Na^+^ content in shoot and root of *Panicum virgatum* L., and enhance the enzymatic activities of SOD, POD, and CAT (Wang et al., 2019). Inhibiting ascorbic acid synthesis regulation gene *VTC1-3* by RNA interfering technology could increase ROS production and aggravate salt damages in rice roots (Wang et al., 2018).

In the present study, rhizosphere salt stress caused root cell damage and irregular cell morphology at the subcellular level. Exogenous ALA application *via* leaf spraying repaired the cell wall and alleviated the degree of its thickening. These results are consistent with those of a study on *B. napus* L., where ALA application repaired the morphological disorders of root cells caused by Cd stress (Ali et al., 2013a). In addition, the number of mitochondria in apical cells under salt stress can be increased by exogenous ALA. On the other hand, the number of mitochondria in the root tip cells increased under excessive KNO_3_ stress (Čiamporová and Mistrík, 1993). Mitochondria are the energy-producing organs in plant cells. The increase in mitochondria can reveal a “mitochondrial pump” function, and develop the intracellular energy regulating mechanism, which ensures energy supply during the adaptation to salt stress. Therefore, the increase in the mitochondrial number is an adaptation mechanism of root cells to stress conditions. Exogenous ALA promotes an increase in the mitochondrial number and function in apical cells to produce more energy to and thus further adapt to salt stress. In addition, the ion concentration in the cytoplasm increased under salt stress, which led to deposition; however, exogenous ALA reduced the cytosolic concentration. Furthermore, after ALA treatment, the ion deposition in vacuoles was more obvious under NaCl, which verified that exogenous ALA application enhanced the ion compartmentalization capacity of root tip cells in cucumber seedlings.

## Conclusion

Salt stress significantly inhibited the root architecture and activity of cucumber seedlings. However, the adverse effects caused by NaCl were shown to be abrogated by exogenous ALA application. On the one hand, the application of ALA improved the excretion of Na^+^ from the cytoplasm by upregulating the H^+^-ATPase and Na^+^/H^+^ antiporter on the plasma membrane. On the other hand, the H^+^-ATPase and Na^+^/H^+^ antiporter on the tonoplast could also be enhanced at both the transcriptional and translational levels to compartmentalize Na^+^ into the vacuoles. Therefore, the ultrastructure of apical cells and the biochemical reactions in the cytoplasm could be ameliorated by exogenous ALA, to effectively alleviate the effects and damage caused by salt stress in cucumber roots and shoots.

## Data Availability Statement

The original contributions presented in the study are included in the article/supplementary material; further inquiries can be directed to the corresponding author.

## Author Contributions

YW, ZT, and JY conceived and designed the research. YW, XX, and NL conducted the experiments. YW, LH, and JL analyzed the data and prepared the figures and illustrations. YW wrote the main body of the manuscript. WL, AC-U, and JX read the manuscript and made valuable inputs. All authors contributed to the article and approved the submitted version.

### Conflict of Interest

The authors declare that the research was conducted in the absence of any commercial or financial relationships that could be construed as a potential conflict of interest.
